# Influence of exposure of customized dental implant abutments to different cleaning procedures: an in vitro study using AI-assisted SEM/EDS analysis

**DOI:** 10.1186/s40729-023-00498-8

**Published:** 2023-09-20

**Authors:** Paul Hofmann, Andreas Kunz, Franziska Schmidt, Florian Beuer, Dirk Duddeck

**Affiliations:** 1 Department of Oral Diagnostics, Digital Health and Health Services Research, Charité-Universitätsmedizin Berlin, Corporate Member of Freie Universität Berlin, Humboldt-Universität Zu Berlin, Aßmannshauser Str. 4-6, 14197 Berlin, Germany; 2 Private Dental Laboratory, Schumannstraße 1, 10117 Berlin, Germany; 3 Department of Prosthodontics, Geriatric Dentistry and Craniomandibular Disorders, Charité-Universitätsmedizin Berlin, Corporate Member of Freie Universität Berlin, Humboldt-Universität Zu Berlin, Aßmannshauser Str. 4-6, 14197 Berlin, Germany; 4 Research Department, CleanImplant Foundation, Pariser Platz 4a, 10117 Berlin, Germany

**Keywords:** Two-piece abutment, CAD/CAM, Contamination, Cleaning methods, Scanning electron microscopy, Energy-dispersive X-ray spectroscopy, SEM–EDS analysis, Disinfection, Machine learning, Segmentation

## Abstract

**Purpose:**

Dental implant abutments are defined as medical devices by their intended use. Surfaces of custom-made CAD/CAM two-piece abutments may become contaminated during the manufacturing process in the dental lab. Inadequate reprocessing prior to patient care may contribute to implant-associated complications. Risk-adapted hygiene management is required to meet the requirements for medical devices.

**Methods:**

A total of 49 CAD/CAM-manufactured zirconia copings were bonded to prefabricated titanium bases. One group was bonded, polished, and cleaned separately in dental laboratories throughout Germany (LA). Another group was left untreated (NC). Five groups received the following hygiene regimen: three-stage ultrasonic cleaning (CP and FP), steam (SC), argon–oxygen plasma (PL), and simple ultrasonic cleaning (UD). Contaminants were detected using scanning electron microscopy (SEM) and energy-dispersive X-ray spectroscopy (EDS) and segmented and quantified using interactive machine learning (ML) and thresholding (SW). The data were statistically analysed using non-parametric tests (Kruskal–Wallis test, Dunn’s test).

**Results:**

Significant differences in contamination levels with the different cleaning procedures were found (*p* ≤ 0.01). The FP–NC/LA groups showed the most significant difference in contamination levels for both measurement methods (ML, SW), followed by CP–LA/NC and UD–LA/NC for SW and CP–LA/NC and PL–LA/NC for ML (*p* ≤ 0.05). EDS revealed organic contamination in all specimens; traces of aluminum, silicon, and calcium were detected.

**Conclusions:**

Chemothermal cleaning methods based on ultrasound and argon–oxygen plasma effectively removed process-related contamination from zirconia surfaces. Machine learning is a promising assessment tool for quantifying and monitoring external contamination on zirconia abutments.

**Graphical Abstract:**

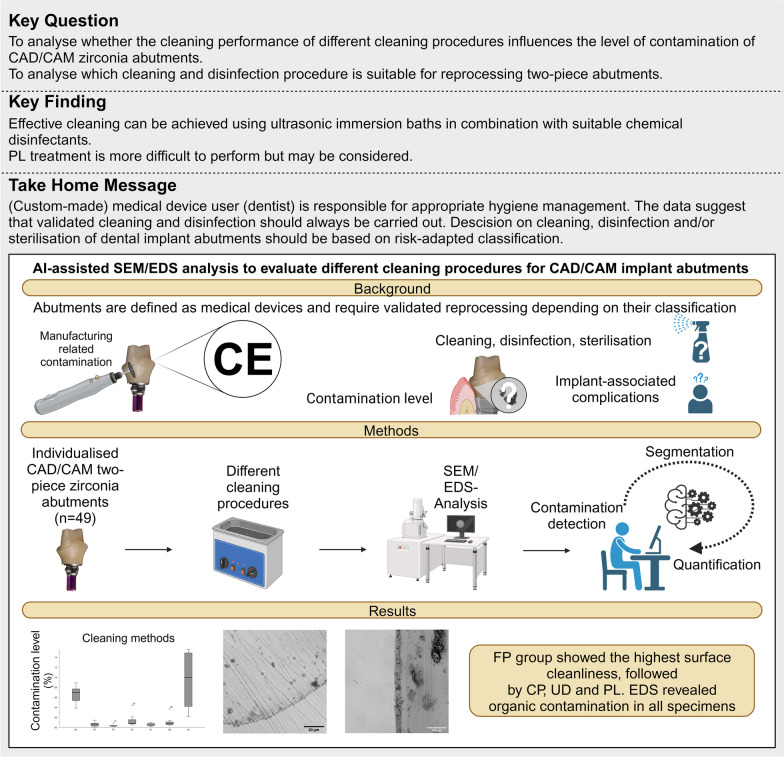

## Background

Implant abutments in the dental implant system are the connecting element between implant-supported prostheses/crowns and endosseous implant bodies. This titanium or all-ceramic foreign body remains permanently in direct contact with the peri-implant mucosa in a critical and bacteria-rich environment between the peri-implant bone and the oral cavity as a transgingival support for the artificial tooth or implant-supported prosthesis. As the barrier function of the peri-implant soft tissue collar is inferior to that of natural teeth, recent research has increasingly focused on improving the quality of the peri-implant soft tissue interface, especially regarding cleaning procedures before placement [1–11].

To address the requirements of aesthetically sensitive restoration areas, customised all-ceramic CAD/CAM abutments are increasingly used to achieve an anatomically correct emergence profile. Two-piece implant abutments consisting of a CAD/CAM-manufactured zirconia coping bonded to a premanufactured titanium base are currently the first choice for demanding restorations. This is due to their excellent biocompatibility, the improved material properties with precise fit at the implant–abutment interface, and the improved tooth-coloured masking of the underlying titanium implant [8, 12–16]. Nowadays, CAD/CAM production is carried out in a central milling facility, a dental lab, or with an in-office milling machine [17]. However, implant superstructures can become contaminated during manufacturing, transport, and packaging [18, 19]. In particular, customized two-piece abutments have shown higher contamination levels after reprocessing than one-piece abutments [2, 5]. According to several laws, regulations and recommendations, implant abutments are classified by manufacturers as medical devices based on their intended use [20–22]. Dental practitioners use custom-made medical devices on a daily basis. They need to be aware of the risk-adapted classification to perform appropriate hygiene management before implant abutment placement. As defined in the European Medical Devices Regulation (MDR EU 2017/745), dental implant abutments are:

- intended for long-term use
- an invasive device
- an implantable device

Such devices are classified according to the MDR classification rules as follows: *“class IIb if they are intended for long-term use, except if they are used in the oral cavity as far as the pharynx, in an ear canal up to the ear drum or in the nasal cavity and are not liable to be absorbed by the mucous membrane, in which case they are classified as class IIa”* [20]. This classification is also consistent with the FDA’s medical device regulations [Code of Federal Regulations (CFR): 21 CFR 872.3630] [23].

Risk-adapted reprocessing (cleaning, disinfection, and, if required, sterilisation) can be derived from medical device classification [21, 22]. To this end, dentists now have unprecedented reprocessing strategies with appropriate substances and instruments [24]. However, the cleaning efficacy of cleaning and disinfection procedures for custom-made implant prosthetic components has yet to be sufficiently scientifically investigated. This is due in part to the lack of tools to quantitatively measure and monitor process-related contamination before and after different cleaning procedures and in part to the fact that, according to the current state of research, there is a lack of knowledge about the exact clinical risks of contaminated implantable devices. Binding limit values for acceptable contamination of medical devices still need to be added [25, 26].

This study aimed to determine the level of contamination on zirconia surfaces at the mucosal transition zone between the oral cavity and peri-implant bone after fabrication and reprocessing. Second, identify suitable cleaning and disinfection procedures for reprocessing customized all-ceramic abutments. The null hypothesis to be tested and confirmed was that the mean values did not differ significantly between the different cleaning methods.

## Methods

### Sample preparation and cleaning procedures

**Fig. 1:**
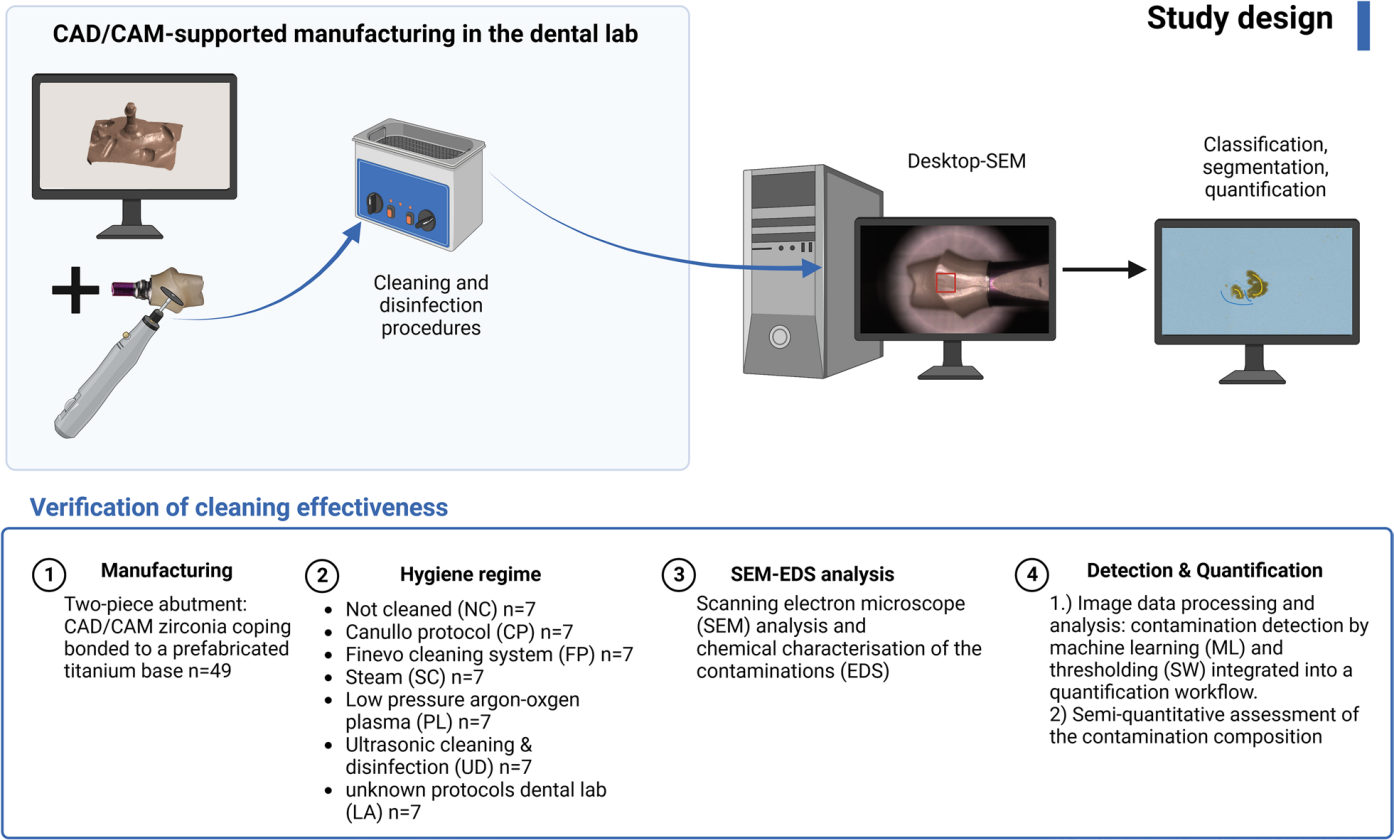
Study design shows the number of cleaning groups and the evaluation process. Created with BioRender.com

**Fig. 2:**
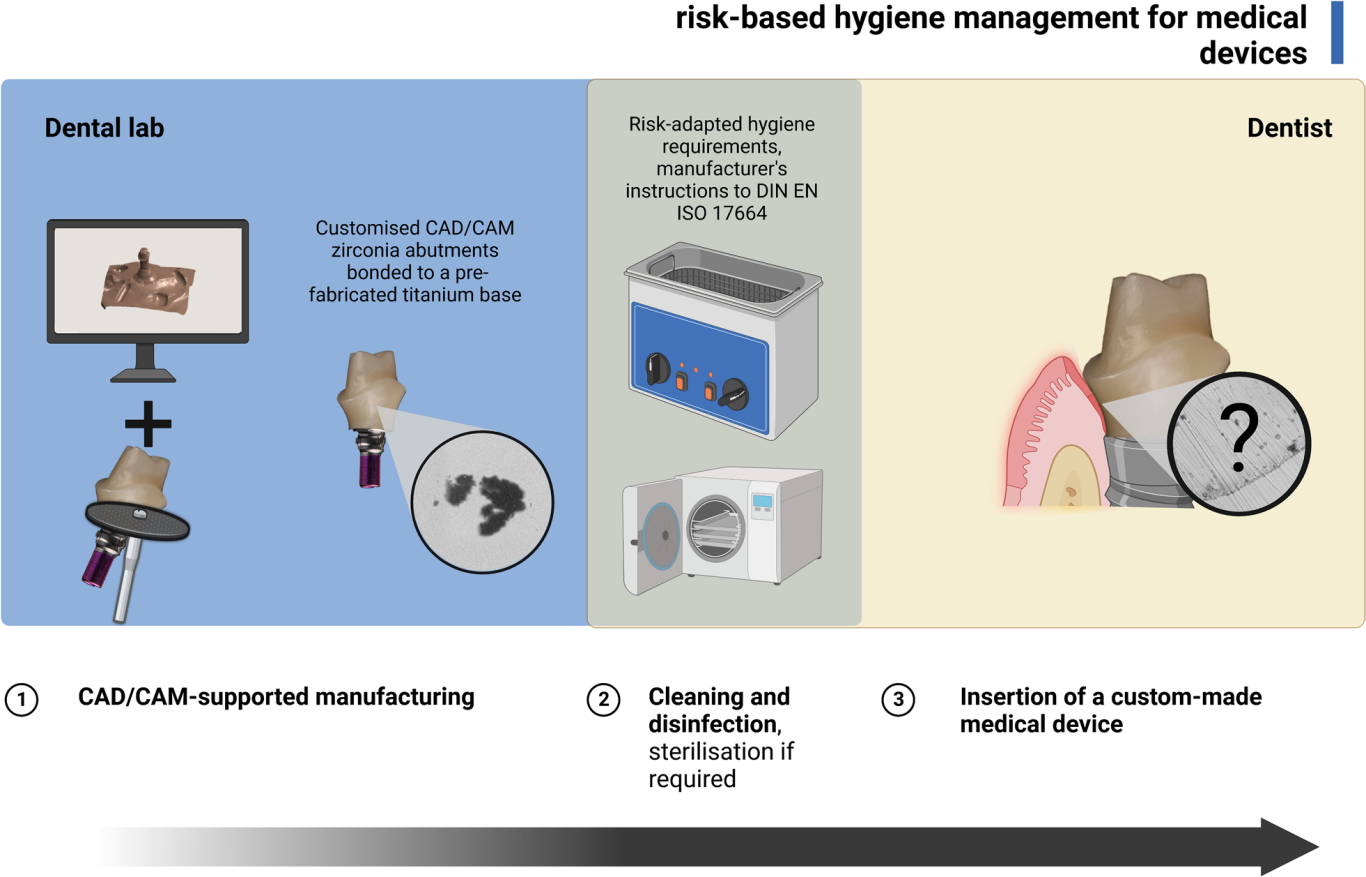
CAD/CAM manufacturing, reprocessing, and insertion of dental implant abutments. Created with BioRender.com

In this study, 49 customised two-piece implant abutments were fabricated in a dental laboratory. Based on a patient case for the implant-retained single crown in FDI position 14, an all-ceramic CAD/CAM zirconia coping (Anatomic coloured A2, Zirkonzahn, Gais, Italy) was bonded to a prefabricated titanium base (Titanium base CAD/CAM, 4.3 mm diameter, CAMLOG Biotechnologies AG, Basel, Switzerland). The external geometry had a prosthetic height of 8.20 mm and an abutment shoulder width of 5.70 mm. The two-piece zirconia abutments were randomly divided into seven study groups of seven specimens each (Fig. 1). In group LA, an unopened premanufactured titanium base, an already milled zirconia abutment, and the resin cement (Multilink Hybrid Abutment, Ivoclar Vivadent, Schaan, Liechtenstein) were sent to seven dental laboratories throughout Germany with the instruction to return the two-piece abutments cleaned according to their protocol and ready for clinical use. It was unclear how the laboratories performed cleaning and disinfection. The bonding surfaces of the 42 remaining two-piece titanium base and all-ceramic copings were blasted with aluminum oxide particles (Al2O3) 50 µm in size (Cobra 50 µm, Renfert, Hilzingen, Germany) at a reduced pressure of 0.8 bar and a distance of 10 mm in the dental laboratory (spot blasting unit P-G 400, Hanisch + Rieth, Winterbach, Germany) [27]. The surfaces were then steamed for 30 s and dried with oil-free air. The bonding surfaces were marked with a permanent marker before sandblasting [28]. The surfaces were sandblasted until the colour was removed entirely. The titanium base and zirconia abutment cleaned bonding surfaces were conditioned (Monobond plus, Ivoclar Vivadent, Schaan, Liechtenstein) and bonded with resin cement according to the manufacturer's instructions (Multilink Hybrid Abutment, Ivoclar Vivadent). Subsequently, the excess cement was removed, and the specimens were polished in two steps using ceramic polishers with diamond grit (94003C and 94003M, Gebr. Brasseler/Komet Dental, Lemgo, Germany) [29]. After the final surface treatment, specimens were randomly grouped into seven pieces and subjected to six different hygiene regimes: no cleaning (NC), multi-stage ultrasonic cleaning with various disinfectants (CP, FP), steam cleaning (SC), low-pressure argon–oxygen plasma cleaning (PL) and single-stage ultrasonic cleaning and disinfection (UD) (full description in Table 1). The hygiene management in the laboratory-based group (LA) was not subsequently queried and remains unclear. After cleaning, all specimens were individually sealed in sterilisation pouches (HS-Sterifoil, Henry Schein Dental, Langen, Germany) (Fig. 2). The materials used in this study are listed in Table 2.

**Table 1:** Cleaning and disinfection methods used in this study

Group	Cleaning method	Cleaning solutions and Devices, respectively, Manufacturer
NC	The samples were dried with oil-free air without further cleaning. Uncleaned abutments served as the control group	–
CP	Three-stage cleaning process in an ultrasonic bath at 37 kHz, all grouped samples were immersed successively for 10 min at 60 °C: pure acetone, pure ethanol, and an antibacterial solution. The samples were stored in distilled water at 60 °C for 5 min between the ultrasonic immersions. In total, the cleaning process took 45 min [2]	SONICA CL4% Easyclean, Renfert, Hilzingen, Germany
FP	Three-stage cleaning process in an ultrasonic bath at 40 kHz. The two-piece abutments were immersed for 5 min each at 30 °C in three different solutions. The first solution contained an industrially prefabricated cleaning liquid with a disinfecting effect, followed by 80% ethanol and purified water [5]	Finevo cleaning system Finevo Ultrasonic, bredent GmbH & Co. KG, Senden, Germany
SC	Steam cleaning for 30 s with 4 bars at 158 °C and 10 cm distance to the cleaning object	Distilled water Wasistream Classic II, Wassermann, Hamburg, Germany
PL	A low-pressure plasma unit conducted the cleaning with a high-frequency generator of 100 kHz/200 W. The samples were individually screwed into an individualised sample holding device and positioned centrally in the vacuum chamber. The automatic program was set according to the manufacturer’s recommendations: process gases argon and oxygen in the same mixing ratio via mass flow controller (MFC), 0.20 mbar set pressure, a set temperature of 70 °C, and reduced power of 80%. The treatment lasted 22 min, with an effective plasma treatment of 20 min	Argon and oxygen gas Femto PCCE Zahntechnik, Diener Electronic, Ebhausen, Germany
UD	Ultrasonic cleaning at 37 kHz with disinfecting solution for 5 min at 30 °C and subsequent rinsing with sterile water	MD 520 Impression Disinfection Ampuwa Easyclean, Renfert, Hilzingen, Germany
LA	All dental labs have been instructed to supply the two-piece abutment ready for patient care. The method used to clean and disinfect the two-piece abutments was deliberately left in the dark	Unknown

**Table 2:** Materials used in this study

Materials	Composition	Manufacturer	Article-no	Lot-no
Titanium base Ø 4.3 mm	Titanium alloy Ti6Al4V, Titan 90%, aluminium 6%, vanadium 4%	CAMLOG Biotechnologies AG	K2244.3848	110088310
Zirkonzahn anatomic coloured A2	ZrO_2_ main component, Y_2_O_3_ 4–6%, Al_2_O_3_ < 1%, SiO_2_ < 0.02%, Fe_2_O_3_ < 0.01%, Na_2_O < 0.04%	ZIRKONZAHN GMBH	14 ZRHB8021A01	ZB923OA
Multilink Hybrid Abutment Cement	Base: Ytterbiumtrifluorid, Bis-EMA, Bis-GMA, 2-Hydroxy-ethyl methacrylate, 2-Dimethylaminoethylmethacrylat Catalyst: Ytterbiumtriflourid, Bis-EMA, Urethandimethacrylat, 2-Hydroxyethylmethacrylat, Dibenzoylperoxid	Ivoclar Vivadent	638959AN	Z01N30
Lab analogue Ø 4.3 mm	Titanium alloy Ti6Al4V, Titan 90%, aluminium 6%, vanadium 4%	CAMLOG Biotechnologies AG	K3010.4300	20093898
Monobond plus	Ethanol, silane, methacrylate phosphoric ester	Ivoclar Vivadent	638959AN	Z028SW
Ceramic polisher	20–50% polyurethane and/or silicone, 40–80% abrasive particles, 0–10% color pigments	Gebr. Brasseler GmbH & Co. KG	94003C, 94003M	519801
Acetone	> 95% Acetone	Fisher Scientific U.K. Limited	16120	1725630
Ethanol	Ethanol 96%	Dr. K. Hollborn & Söhne	200-578-6	0219
SONICA CL4%	100 g Sonica CL 4% contains 15 g Cetrimide, 1,5 g Chlorhexidine gluconate, Excipients: co-formulants, fragrance, colouring, and purified water up to 100,0 g	SOLTEC S.r.l	090.005.0017	J1523
FINEVO CLEANING SYSTEM FINEVO 01 Starter-Set	FINEVO 01.1: cleaning fluid with disinfecting effect	bredent GmbH & Co. KG	53001001	496262
	FINEVO 01.2: 80% Ethanol	bredent GmbH & Co. KG	53001002	496263
	FINEVO 01.3: highly purified water (aqua bidestillata)	bredent GmbH & Co. KG	53001003	496264
MD520	Aldehydes, quaternary ammonium compounds, alcohols, non-ionic surfactants, complexing agents, and auxiliaries in aqueous solution	DÜRR DENTAL SE	CDA520C6150	1925009
Ampuwa	sterile, pyrogen-free water (Aqua ad iniectabilia)	Fresenius Kabi Deutschland GmbH	1088813	13MMP051
Argon gas	Argon compressed, Capacity 60 L, Volume 950 ml	CFH Löt– und Gasgeräte GmbH	EAN: 4001845525143	–
Oxygen gas	Oxygen compressed, Capacity 120 L, Volume 930 ml	ROTHENBERGER Industrial GmbH	EAN: 4004625357415	–

The data listed correspond to the manufacturer's specifications. Zr02 = zirconium oxide; Y2O3 = yttrium (III) oxide; Al203 = aluminum oxide; Si02 = silicon dioxide; Fe203 = iron (III) oxide; Na20 = sodium oxide; Bis-GMA = bisphenol A diglycidyl methacrylate; Bis-EMA = bisphenol A diglycidyl methacrylate ethoxylated

### Imaging acquisition (SEM) and image data processing and analysis

All specimens were subjected to SEM and EDS analysis (Phenom-World B.V., Eindhoven, The Netherlands). The scanning electron microscope is equipped with a highly sensitive backscattered electron (BSE) detector and operates at an accelerating voltage of 15 kV. Backscattered electron imaging allows conclusions to be drawn about the chemical nature and location of various contaminants on the sample. Low atomic number elements, such as carbon, appear relatively dark, while higher atomic number elements, such as zirconium, appear relatively bright. The high spatial resolution and large field of view of a stitched image allowed us to filter out areas of interest (contaminations) for subsequent EDS analysis. Two open-source programs were used to classify, segment, and quantify the contaminants: *Fiji* (ImageJ, version 1.53c) and *ilastik* (Ilastik, version 1.3.3) [30, 31]. Two in-house developed workflows based on pixel-based machine learning (ML) and thresholding (SW) were compared. A detailed image processing and analysis steps description has already been published [25].

### Semiquantitative chemical surface analysis (EDS)

All specimens were evaluated with EDS point analyses to determine the chemical composition of the contaminations in the predefined observation field. Due to the fully integrated thermoelectrically cooled silicon drift detector (SDD), all measurements were performed in temporal relation to the SEM analysis. At least two different points for each specimen were analysed for elemental composition at 2500 × magnification. The working distance was 6 mm with a field of view (FOV) of 108 µm. Excited by the interactions between the irradiated electrons and the electromagnetic field of the investigated samples, the characteristic X-ray radiation was emitted element-specifically. The accelerating voltage was 15 kV, and the active detector had an area of 25 mm^2^ with a take-off angle (TOA) of 29°. The peak position identifies the element, while the intensity of the signal indicates the number of X-ray quanta detected per element. An automatic peak deconvolution algorithm evaluated the spectrum through the coupled software (Phenom Elemental Identification Version 3.8.4.0, ThermoFisher Scientific, Eindhoven, The Netherlands). For peak identification of the acquired spectrum, we deactivated elements with a lower match below a peak match of ≥ 0.95. The relative concentration fractions of the detected elements (in atomic per cent) were calculated for all groups. Elements ≤ 1 atomic per cent (at. %) were filtered out (Table 3).

### Statistical analysis

Data were analysed using a statistical program (SPSS version 27.0, IBM SPSS). Nonparametric methods (Kruskal–Wallis test) were used to test the influence of the cleaning method on the level of contamination. Dunn’s test was used for multiple comparisons between cleaning methods and contamination levels. *p* values for subsequent multiple comparisons were corrected according to the Bonferroni method. The effect size was measured using Cohen’s d. Descriptive data analysis was used to quantitatively assess contamination levels and EDS analysis. All statistical tests were performed at a two-sided significance level of α = 0.05 (Fig. 2).

## Results

**Fig. 3:**
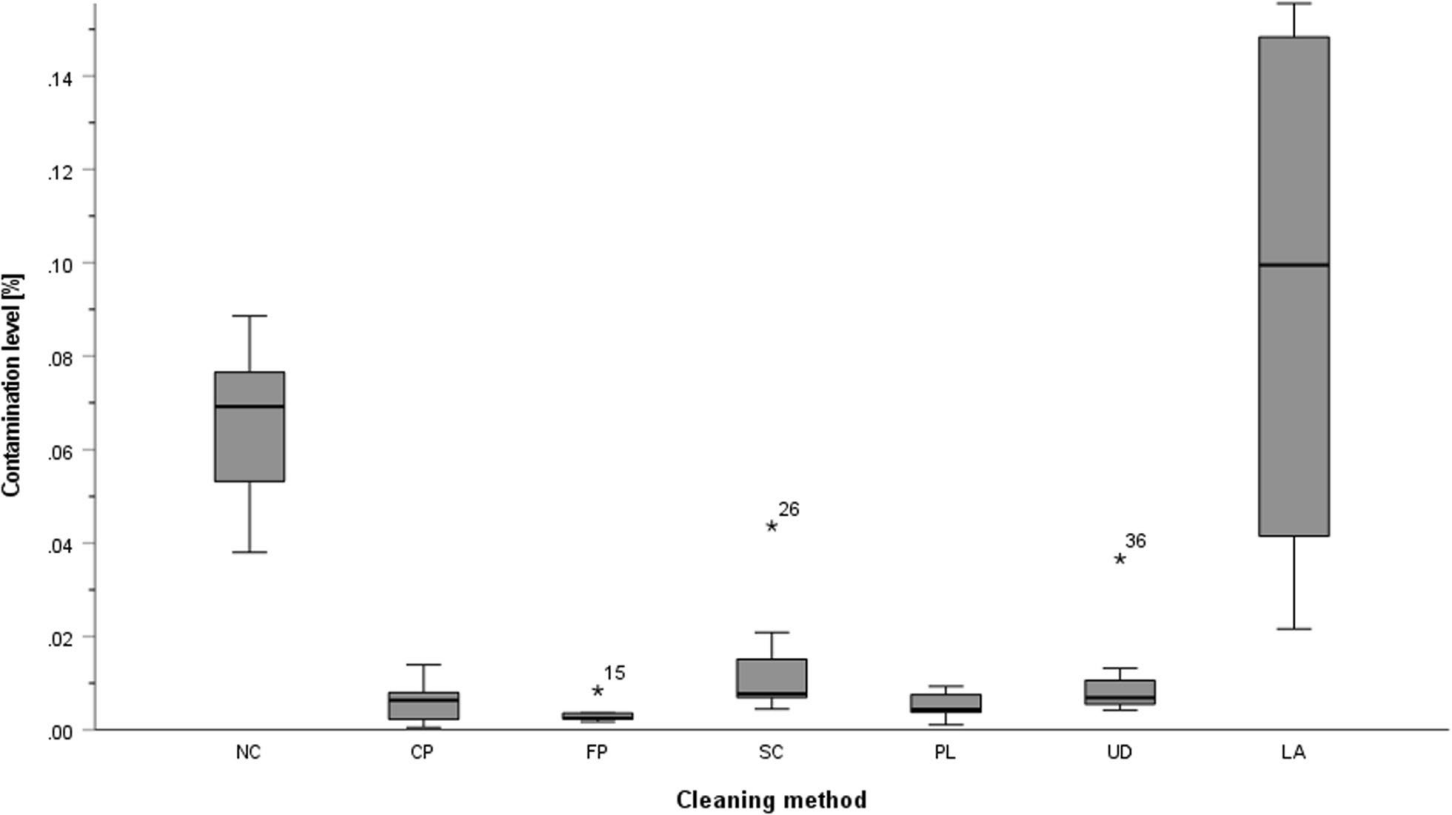
Box-plot diagram of the contamination level in per cent for ML. One outlier of 0.31% in the LA group has been removed for clarity

**Fig. 4:**
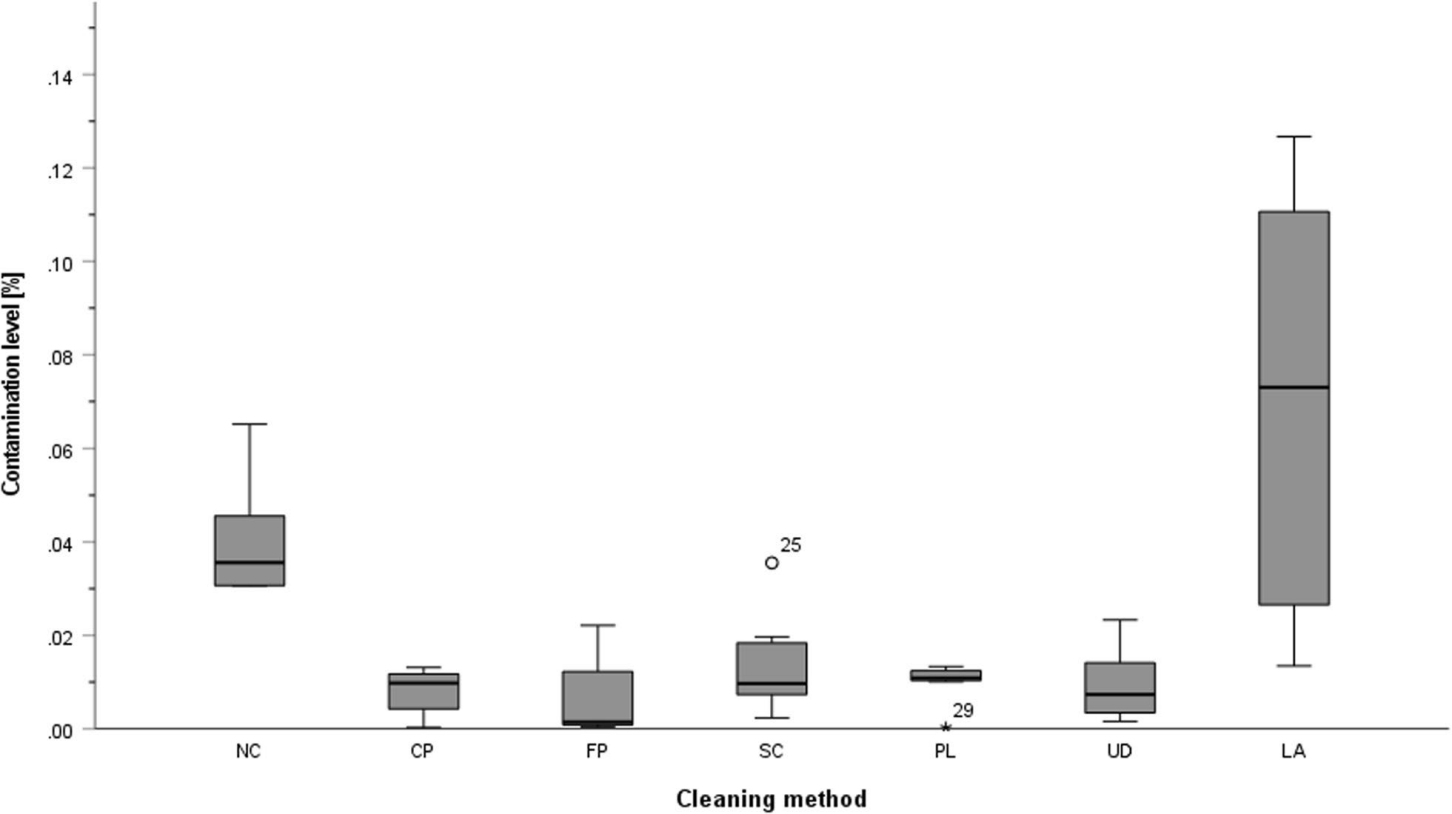
Box-plot diagram of the contamination level in per cent for SW. One outlier of 0.29% in the LA group has been removed for clarity

A summary of the data and statistical analyses is shown in Figs. 3, and 4, Tables 3, 4 and 5.

### Quantification using interactive machine learning

The contamination level of specimens cleaned by the FP (minimum 0.0018%; median 0.0025%; maximum 0.0084%), CP (minimum 0.0005%; median 0.0064%; maximum 0.0140%) and PL (minimum 0.0011%; median 0.0044%; maximum 0. 0093%) showed the lowest residual contamination compared to the UD (minimum 0.0042%; median 0.0069%; maximum 0.0367%), SC (minimum 0.0045%; median 0.0077%; maximum 0.0437%), NC (minimum 0.0380%; median 0.0692%; maximum 0.0886%) and LA (minimum 0.0216%; median 0.0995%; maximum 0.3089%) methods (Fig. 3). A Kruskal–Wallis test indicated significant differences between contamination levels and cleaning methods [H(6) = 33.8, *p* ≤ 0.00001]. Subsequent post hoc tests (Dunn–Bonferroni tests) showed the greatest difference in contamination levels for the FP–LA/NC groups, followed by CP/LA, PL/LA, CP/NC and PL/NC (*p* ≤ 0.05, UD/LA, UD/NC, SC/LA, FP/SC, SC/NC, FP–CP/PL/UD, CP–PL/UD/SC, PL–UD/SC, UD/SC, NC/LA were not significantly different) (Table 3). All significant group comparisons had a large effect size according to Cohen’s d. The most significant effect was seen in the FP/NC, PL/NC and CP/NC group comparisons with d = 4.92, d = 4.75 and d = 4.60, respectively (Fig. 3, Table 3).

**Table 3:** Statistical analysis of the cleaning method’s influence on the cleaning efficiency for method ML

Group 1–Goup 2	M_Diff_ in %	*d*_*Cohen*_	P	adj. P^a^
FP–CP	− 0.0024		0.421	1
FP–PL	− 0.0019		0.379	1
FP–UD	− 0.0079		0.089	1
FP–SC	− 0.0108		0.033	0.693
FP–NC	− 0.0616	4.92	0.000	**0.001**
FP–LA	− 0.1122	1.57	0.000	**0.000**
CP–PL	0.0006		0.940	1
CP–UD	− 0.0055		0.369	1
CP–SC	− 0.0084		0.184	1
CP–NC	− 0.0591	4.60	0.001	**0.015**
CP–LA	− 0.1098	1.54	0.000	**0.010**
PL–UD	− 0.0061		0.411	1
PL–SC	− 0.0089		0.210	1
PL–NC	− 0.0597	4.75	0.001	**0.020**
PL–LA	− 0.1103	1.55	0.001	**0.013**
UD–SC	− 0.0029		0.667	1
UD–NC	− 0.0536		0.013	0.270
UD–LA	− 0.1043		0.009	0.196
SC–NC	− 0.0508		0.040	0.832
SC–LA	− 0.1014		0.030	0.631
NC–LA	− 0.0506		0.911	1

Decimals are rounded. Kruskal–Wallis test was followed by post hoc tests (a = P values were corrected according to Bonferroni correction with a factor of 21 for multiple tests). The significance level was 0.05. The bold type indicates statistically significant differences. The effect size (d_Cohen_) was calculated from the mean difference and the pooled standard deviations. NC = not cleaned; CP = cleaning protocol according to Canullo; FP = cleaning protocol according to FINEVO CLEANING SYSTEM; SC = steam cleaning; PL = low-pressure plasma cleaning; UD = ultrasonic cleaning and disinfection; LA = laboratory group with unknown cleaning methods

### Threshold-based quantification

The contamination level of specimens cleaned by the CP (minimum 0.0003%; median 0.0098%; maximum 0.0132%), FP (minimum 0.0004%; median 0.0015%; maximum 0.0221%) and UD (minimum 0.0016%; median 0.0074%; maximum 0. 0.0233%) was lower than for specimens cleaned by the SC (minimum 0.0024%; median 0.0097%; maximum 0.0355%), PL (minimum 0.0003%; median 0.0109%; maximum 0.0134%), NC (minimum 0.0305%; median 0.0356%; maximum 0.0652%) and LA (minimum 0.0135%; median 0.0730%; maximum 0.2895%) methods (Fig. 4). A Kruskal–Wallis test showed that the level of contamination was influenced by the cleaning methods [H(6) = 28.2, *p* ≤ 0.0001]. Subsequent post hoc tests (Dunn–Bonferroni tests) showed the best decontamination for the FP method, followed by CP and UD (*p* ≤ 0.05, FP–CP/UD/PL/SC, CP–UD/PL/SC, UD–PL/SC, PL–SC/NC/LA, SC–NC/LA and NC/LA were not significantly different) (Table 4). All significant group comparisons had a large effect size according to Cohen’s d. The CP/NC and FP/NC group differences had the most significant effect size with d = 3.36 and d = 3.00, respectively (Fig. 4, Tables 4, 5).

**Table 4:** Statistical analysis of the cleaning method’s influence on the cleaning efficiency for method SW

Group 1–Group 2	M_Diff_ in %	*d*_*Cohen*_	P	adj. P^a^
FP–CP	− 0.0007		0.708	1
FP–UD	− 0.0025		0.600	1
FP–PL	− 0.0029		0.379	1
FP–SC	− 0.0070		0.246	1
FP–NC	− 0.0334	3.00	0.000	**0.006**
FP–LA	− 0.0858	1.26	0.000	**0.006**
CP–UD	− 0.0017		0.881	1
CP–PL	− 0.0021		0.614	1
CP–SC	− 0.0063		0.432	1
CP–NC	− 0.0326	3.36	0.001	**0.024**
CP–LA	− 0.0850	1.25	0.001	**0.022**
UD–PL	− 0.0004		0.722	1
UD–SC	− 0.0045		0.525	1
UD–NC	− 0.0309	2.81	0.002	**0.040**
UD–LA	− 0.0833	1.22	0.002	**0.038**
PL–SC	− 0.0041		0.779	1
PL–NC	− 0.0305		0.006	0.125
PL–LA	− 0.0829		0.006	0.118
SC–NC	− 0.0264		0.014	0.285
SC–LA	− 0.0788		0.013	0.270
NC–LA	− 0.0524		0.985	1

Decimals are rounded. Kruskal–Wallis test was followed by post-hoc tests (a = P values were corrected according to Bonferroni correction with a factor of 21 for multiple tests). The significance level was 0.05. The bold type indicates statistically significant differences. The effect size (dCohen) was calculated from the mean difference and the pooled standard deviations. NC = not cleaned; CP = cleaning protocol according to Canullo; FP = cleaning protocol according to FINEVO CLEANING SYSTEM; SC = steam cleaning; PL = low-pressure plasma cleaning; UD = ultrasonic cleaning and disinfection; LA = laboratory group with unknown cleaning methods

**Table 5:** Descriptive statistics for comparison of measurement methods in per cent

Cleaning method		Threshold method (SW)	Machine learning method (ML)
		Contamination level (%)	Contamination level (%)
Group NC (*n* = 7)	M	0.041	0.065
	SD	0.013	0.018
	Mdn	0.036	0.069
	IQA	0.017	0.025
Group CP (*n* = 7)	M	0.008	0.006
	SD	0.005	0.005
	Mdn	0.010	0.006
	IQA	0.008	0.007
Group FP (*n* = 7)	M	0.007	0.003
	SD	0.009	0.002
	Mdn	0.001	0.003
	IQA	0.017	0.001
Group SC (*n* = 7)	M	0.014	0.014
	SD	0.011	0.014
	Mdn	0.010	0.008
	IQA	0.014	0.014
Group PL (*n* = 7)	M	0.010	0.005
	SD	0.004	0.003
	Mdn	0.011	0.004
	IQA	0.003	0.004
Group UD (*n* = 7)	M	0.010	0.011
	SD	0.009	0.012
	Mdn	0.007	0.007
	IQA	0.017	0.008
Group LA (*n* = 7)	M	0.093	0.116
	SD	0.096	0.101
	Mdn	0.073	0.100
	IQA	0.101	0.148

Decimals are rounded, *N* = 49, M = mean, SD = standard deviation, Mdn = median, IQA = interquartile range. NC = not cleaned; CP = cleaning protocol according to Canullo; FP = cleaning protocol according to FINEVO CLEANING SYSTEM; SC = steam cleaning; PL = low-pressure plasma cleaning; UD = ultrasonic cleaning and disinfection; LA = laboratory group with unknown cleaning methods

## EDS analysis

**Fig. 5:**
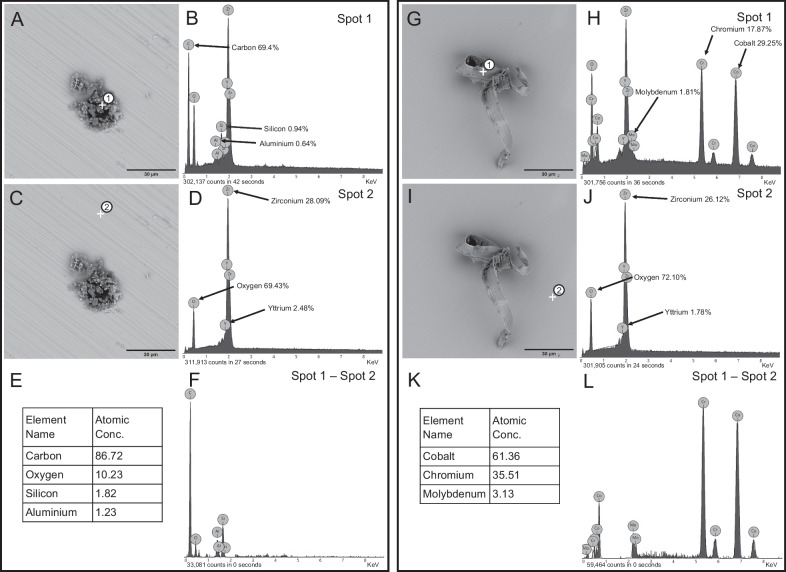
Examples of the elemental composition of the different contaminants and the background composition (zirconia) with Phenom ProX–SEM–EDS; measuring points are marked with a cross and number, percentages in atomic per cent: **A–F** sample from group NC, **B** shows the EDS spectrum for spot analysis spot 1, **D** shows the EDS spectrum for spot analysis spot 2, **E, F** subtracted spectra of the sample from group NC: spectrum 1–spectrum 2; **G–L** sample from group LA, **H** shows the EDS spectrum for spot analysis spot 1,** J** shows the EDS spectrum for spot analysis spot 2, **K, L** subtracted spectra for the sample from group LA: spectrum 1–spectrum 2

**Fig. 6:**
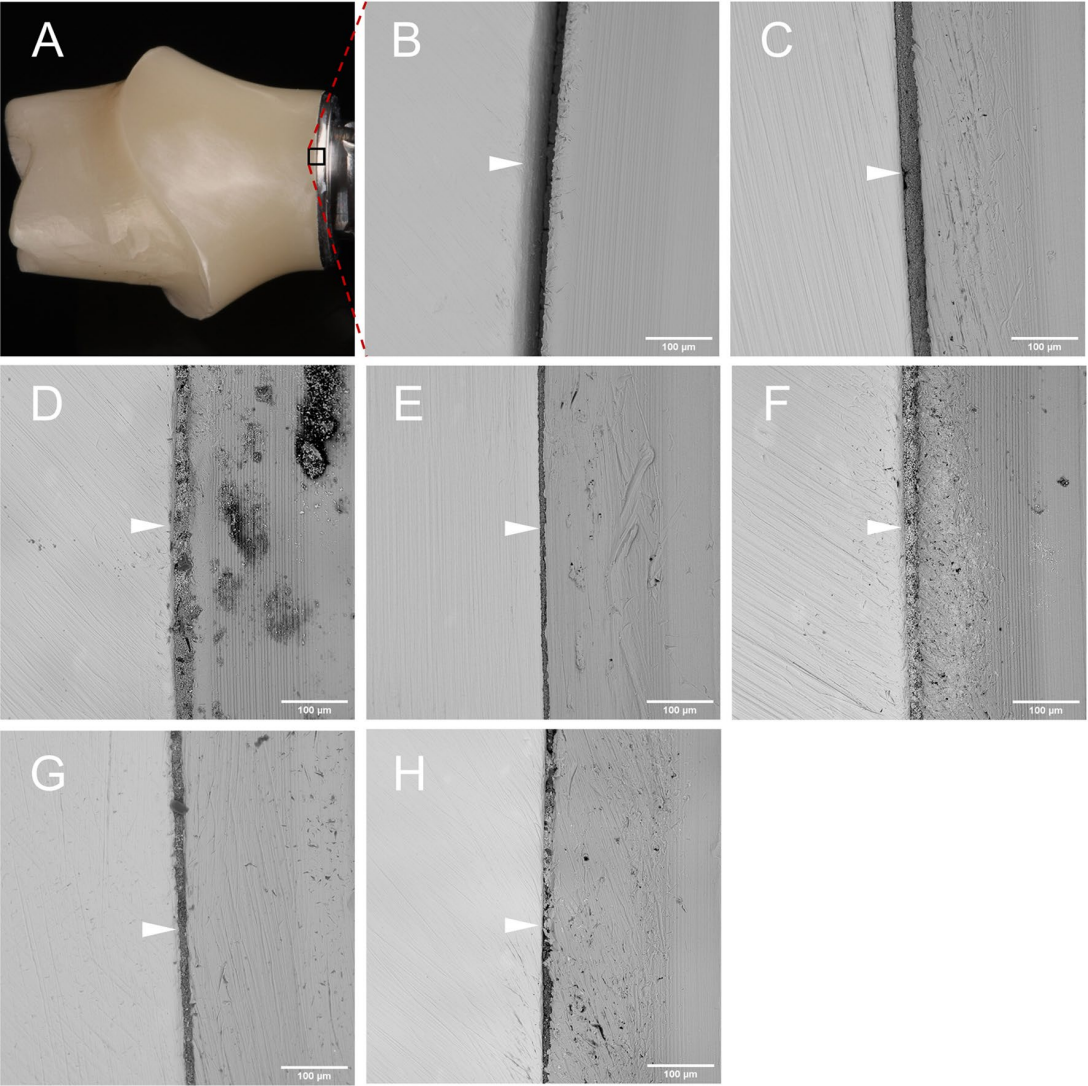
Scanning electron micrographs of the adhesive joint after various cleaning and disinfection procedures (500 × magnification, 537 µm field of view), white arrows mark the adhesive joint: **A** camera overview image, square area selection at the interface between zirconia abutment, adhesive joint, and titanium base, **B** group CP, **C** group FP, **D** group NC, **E** group SC, **F** group PL, **G** group LA, **H** group UD

The elemental analysis of all cleaning groups by EDS point analysis is summarized in Table 6. The presence of four major elements was detected in all observation areas of all cleaning groups: carbon (C), oxygen (O), zirconium (Zr) and yttrium (Y), the latter three being associated with the base material of the samples. Traces of aluminum, silicon and iron were also detected, with a maximum value of iron of 33.3 at. %, followed by aluminum at 25.9 at. % and silicon at 17.5 at. %. Differential EDS was used to separate the received X-ray quanta of the surface contaminants from the signal of the subsurface material. Therefore, the signals of the uncontaminated abutment material were subtracted from the mixed signal of the surface contamination. A representative elemental analysis of contamination spots using SEM–BSE is shown in Fig. 5. These included a contaminant spot from the NC and LA groups (full results in Table 6). The subtracted spectrum shows an organic composition of two major elements: carbon (C) and oxygen (O), with values above 10 at. % (Fig. 5A–F) and an inorganic composition consisting of a metal alloy rich in cobalt (Co) and chromium (Cr) (Fig. 5G–L). To visually check for alterations of the interfaces between the zirconia coping and the titanium base after hygiene management, representative scanning electron micrographs of the adhesive joint from all study groups were presented at 500 × magnification (Fig. 6).

**Table 6:** Descriptive statistics of the EDS analysis

Localisation	Element name	Element symbol	N_P_/N_total_	Mean	Minimum	Maximum	
Point analysis contaminations	Carbon	C	*39/49*	40.5	0	86.1	
	Nitrogen	N	8/49	2.6	0	22.9	
	Oxygen	O	48/49	42.1	0	82.3	
	Fluorine	F	3/49	1.5	0	30.7	
	Sodium	Na	5/49	0.2	0	3.9	
	Aluminium	Al	13/49	2.1	0	25.9	
	Silicon	Si	17/49	2.8	0	17.5	
	Phosphorus	P	1/49	0.2	0	10.1	
	Sulphur	S	8/49	1.1	0	11.2	
	Chlorine	Cl	5/49	0.7	0	13.9	
	Potassium	K	4/49	0.5	0	10.2	
	Calcium	Ca	13/49	1.2	0	8.6	
	Titanium	Ti	6/49	1.1	0	29.3	
	Chrome	Cr	3/49	0.5	0	17.9	
	Iron	Fe	2/49	1.1	0	33.3	
	Cobalt	Co	1/49	0.6	0	29.3	
	Yttrium	Y	29/49	0.9	0	2.4	
	Zirconium	Zr	48/49	10.7	0	25.3	
Point analysis background	Oxygen	O	49/49	65.5	50.9	72.3	
	Yttrium	Y	49/49	2.8	1.9	4.6	
	Zirconium	Zr	49/49	31.7	25.3	44.5	

Decimals are rounded. All data in atomic per cent (at. %). P = Number of positive point analyses for the respective element related to all specimens

## Discussion

Implant abutments are in direct contact with the peri-implant mucosa. The medical device user (dentist) is responsible for risk management before reprocessing and patient care (Fig. 2). Decision-making depends on the type of application; the dentist must classify customised CAD/CAM-manufactured abutments as risk-adapted, considering the manufacturer’s specifications (DIN EN ISO 17664) [21, 22]. As customised implant abutments are often used for late implant-prosthetic rehabilitation after wound healing is complete, a semi-critical classification is usually sufficient [20]. If there is doubt about the classification, the more critical classification should be used [20, 21]. Hygiene management for semi-critical devices must include validated cleaning and disinfection and, if classified as critical, sterilisation prior to patient care. Dentists must also consider appropriate packaging systems and device transportation, as recontaminating the product is possible [18, 20]. However, following the manufacturing process in the dental laboratory, contaminants such as carbon, titanium and aluminum microparticles have been found on the abutment surface adjacent to the mucosal transition zone, even after reprocessing [2, 5, 18]. In addition, customised implant abutments appear to have higher levels of contamination than prefabricated ones [2, 5]. To date, no cleaning and disinfection method has completely removed process-related contamination. The issue of valid reprocessing of implant abutments continues to be debated [32–35]. However, the method for monitoring and measuring decontamination has yet to be researched [25]. When reviewing the literature, no established “gold standard” reference method exists. To date, only a few studies have been identified that have quantified contamination on implant abutments. Contamination detection has so far relied on manual annotation of SEM images by experts [2, 5, 19, 36].

In the present in vitro study, interactive machine learning was used for the first time for classification and segmentation compared to segmentation by thresholding. In addition, quantification was performed in a simple self-written program [25]. Not all hygiene regimes could reduce contamination levels significantly—there were significant cleaning-specific differences. The highest surface cleanliness was achieved by chemothermal ultrasonic cleaning (CP, FP and UD) and argon–oxygen plasma treatment (PL) (Tables 3, 4). The most significant difference in the contamination level between the FP/NC, CP/NC, UD/NC and PL/NC groups should be highlighted. The results are consistent with Canullo et al. and Gehrke et al., who also reported superior ultrasonic cleaning for residual and bacterial decontamination [2, 5, 37]. The most effective surface cleanliness for ultrasonic cleaning was measured by Canullo et al. at 0.0007%; generally higher contamination levels were measured for two-piece abutments as detected by Gehrke et al., even with multi-stage ultrasonic cleaning, which is also consistent with the current measurements [2, 5]. Nevertheless, the data from this study showed deviations from the measured values of Canullo et al. and Gehrke et al. This could be due to differences in material selection, analysis tools, image data processing, and evaluation. While ultrasonic reprocessing with chemical disinfectants follows national and international regulations, plasma treatment is not currently a validated reprocessing strategy [20, 21, 33]. However, its application to all-ceramic biomaterials has been described [4, 38, 39]. Plasma treatment of zirconia resulted in comparable cell adhesion to ultrasound and disinfection, increased collagen fiber density and stable peri-implant bone level compared to steam cleaning measured on titanium abutments [3, 4, 38]. In addition, the cleaning and disinfecting effect has already been highlighted, with no adverse impact on tensile bond strength [2, 40, 41]. However, the data presented here contradicts the efficiency of plasma cleaning published by Farronato et al. His measurements showed that the use of argon plasma alone was inferior to that of argon plasma in combination with ultrasonic cleaning [36]. Against this contradiction, it can be argued that image segmentation was done by visual grid assessment. Squares containing contaminants were scored positive and compared as a percentage of the total number of squares in the grid. This may have led to an inaccuracy compared to direct contamination detection by supervised machine learning or thresholding [25]. Uncleaned specimens showed similar contamination levels to previous studies of one-piece titanium and two-piece zirconia abutments [5, 19]. Steam cleaning, although commonly used clinically, is not a validated reprocessing method, as discussed by Kern et al. [32]. Although Canullo et al. had shown that thermal cleaning with steam for 30 s reduced residual contamination and a cleaning effect was also observed in the present in vitro study, it was significantly different from cleaning with ultrasound and argon oxygen plasma [19] (Tables 3, 4). This was also reflected at the cellular level in lower cell viability and increased bacterial colonization after steam cleaning compared to ultrasound reprocessing [11, 42]. The LA group revealed a wide range of data with reduced efficacy of final cleaning and disinfection methods. Although the data are limited and should be interpreted with caution due to the small sample size, they illustrate the heterogeneity of approaches to hygiene management between dentists and dental laboratories in Germany. This suggests that adequate reprocessing of semi-critical and critical medical devices, such as implant abutments, needs to be adequately implemented.

While the agreement with previous manual expert annotations supports the reliability of the new AI-assisted detection and quantification method, robust validation on independent data and larger heterogeneous data sets currently needs to be improved [25, 43].

As EDS is only an auxiliary tool for morphological characterisation, the results of EDS analysis must be interpreted with limitations. EDS analysis cannot detect carbonaceous contaminants in the molecular range (e.g., from ambient air or packaging) [44]. However, carbonaceous particles in the micrometre range could be identified, which explains the strong carbon signal in almost all measurements (Table 6). On average, the overlying contaminants consisted of 40.5 at. % carbon and metallic contaminants such as aluminum and titanium were detected in isolated cases (Fig. 5). This is agreed with previous spectroscopic investigations [5, 18, 19, 42]. The EDS point analysis is limited in its quantitative significance, and assigning the results to individual cleaning methods was impossible.

SEM analysis of the adhesive joint of the two-piece specimens revealed irregular pits and inclusions that altered the circular milling grooves. In addition, wear particles were detected qualitatively on all prefabricated titanium bases (Fig. 6). This surface roughness and contamination may be attributed to the final manufacturing processes (airborne particle abrasion (APA), bonding, polishing). Different surface cleanliness levels could be observed visually depending on the cleaning method. The specimens exposed to the CP cleaning protocol with acetone-containing solution showed partial dissolution of the adhesive joint (Fig. 6B). While previous studies on pure titanium surfaces have shown no differences in surface properties, adverse effects on the tensile bond strength of two-piece abutments have not been investigated [45]. Further in vitro studies should follow to clarify this issue, but in the meantime, the use of acetone-containing cleaning solutions for hybrid structures should be avoided.

While the effects of surface topography and chemical composition of zirconia on the peri-implant soft tissue are well-understood and make clinical treatment more predictable, the potential negative impact of residual contamination on zirconia abutments in the implant–abutment interface has not been sufficiently investigated [42, 46–50]. In particular, there is a lack of precise knowledge about possible thresholds for “acceptable levels of contamination” of medical devices and in vivo data on immunological responses to contaminated implanted biomaterials that may disrupt the peri-implant tissue seal over time and promote marginal bone resorption. However, contaminations and remnants of handling and manufacture identified on abutments in this study must be related to the concept of foreign body equilibrium, which Albrektsson et al. established years ago to explain marginal bone loss and disturbed osseointegration [51, 52]. An imbalance of this patient-specific foreign body equilibrium, e.g., by the additional entry of foreign microparticles into the transition zone between soft tissue and bone, could lead to immune-mediated foreign body reactions up to peri-implantitis and bone loss. Further studies are required to investigate the relationship between possible individual input of inflammatory immune processes due to process-related residual contamination in the mucosal transition zone and adverse events in the peri-implant tissue. Considering the practical implications of international hygiene regulations, it can be summarised that the user (dentist) has a duty of care in managing invasive medical devices, e.g., customised implant abutments. In this regard, medical devices should be “*manufactured in such a way as to minimise the risks posed by … particles that may be released from the device, including abrasion, degradation products and processing residues*” [20]. This ensures patient safety and may improve the long-term stability of the implant-supported restoration.

## Conclusions

The following conclusions can be drawn based on the data from this study.

For hygiene management of semicritical medical devices, single or multistage ultrasonic immersion baths in combination with suitable chemical disinfectants can achieve effective decontamination on zirconia surfaces. Reprocessing by plasma treatment is not currently a validated process. Although this study has shown that ultrasound-based systems can achieve comparable decontamination, future studies should verify the results. Steam cleaning is not recommended. Regarding measurement methodology, AI-assisted detection and monitoring of process-related contamination could improve manufacturers’ quality management systems, save expert resources, and overcome subjective variability.

## Data Availability

The data sets used and analysed during the current study are available from the corresponding author on reasonable request.

## Abbreviations

SEM: Scanning electron microscope
EDS: Energy dispersive X-ray spectroscopy
CAD/CAM: Computer-aided design/computer-aided manufacturing
MDR: Medical devices regulation
CFR: Code of Federal Regulations
BSE: Back scattered electrons
SDD: Silicon drift detector
FOV: Field of view
TOA: Take-off angle
